# Medicinal keystone species: linking local medicinal systems and social-ecological systems in a One Health perspective

**DOI:** 10.1186/s13002-026-00887-4

**Published:** 2026-04-05

**Authors:** Michael Rapinski, Washington Soares Ferreira Júnior, Marc-Alexandre Tareau, Guillaume Odonne

**Affiliations:** 1 UAR 3456 LEEISA (CNRS, Université de Guyane, IFREMER), Cayenne, French Guiana; 2 UMR 7206 Eco-Anthropologie (CNRS, MNHN, Université Paris Cité), Paris, France; 3 Universidade de Pernambuco, 55800-000 Nazaré da Mata, PE Brazil; 4 Laboratório de Ecologia e Evolução de Sistemas Socioecológicos (LEA), Departamento de Botânica, Universidade Federal de Pernambuco, Av. Prof. Moraes Rego, Cidade Universitária, PE 50740-600 Recife, Brazil; 5 Cayenne Hospital, CIC INSERM 1424, CHU de Guyane, Cayenne, French Guiana

**Keywords:** Ethnomedicine, Traditional medicine, *Quassia amara*, *Castor canadensis*, Reciprocity, Cultural keystone species

## Abstract

Local medicinal systems, sometimes referred to as “traditional”, are characterized by a strong integration between human, animal and ecosystem health, of which the rich interdependencies are central tenets of the One Health perspective. Drawing inspiration from the concept of cultural keystone species, developed over the last twenty years to deepen understanding of the importance of certain non-human species in local societies and the reciprocal relationships between them, we propose the concept of medicinal keystone species.

They can be defined according to six criteria: (i) intensity, type, and diversity of medicinal use; (ii) naming and terminology within languages; (iii) role in narratives, ceremonies, or symbolism; (iv) persistence and memory of use despite changes in and threats to the medicine system; (v) Visibility and intelligibility of the foundations of a medicinal system; (vi) opportunities for resource acquisition from beyond local territories. Two examples are presented to illustrate the medicinal keystone species framework: (1) *Quassia amara*, a plant prominent in French Guianese Creole medicine, and (2) *Castor canadensis*, an animal central to medicinal systems of Algonquian Peoples of Eastern Canada.

This concept may play an important role in the conservation of social-ecological systems. By identifying sets of resources that fulfil structural and functional roles in both local medicinal systems and the cultural identity of peoples and society, it helps recognize the medicinal knowledge systems of Indigenous Peoples and Local Communities (IPLC), including Afro-descendants, and emphasizes key components of their *materia medica*. In this way, it can support more effective access to local medicines in healthcare systems.

## Science Highlights

Medicinal keystone species are central to local medicinal systems.

Medicinal keystone species can be rigorously identified using 6 criteria.

Medicinal keystone species link health and well-being, culture, and ecosystems.

Protecting medicinal keystone species supports biocultural conservation and local medicinal systems.

## Policy and practice recommendations

• Consider local medicinal systems and their keystone species in healthcare access and planning.

• Recognize local medicinal systems and their keystone species in public health and One Health strategies.

• Support community-led management of local medicinal systems and their keystone species.

• Integrate medicinal keystone species into conservation, sustainable use, and land-use policies.

## History of concepts

Ecologists have long understood that certain species play crucial roles in defining entire ecosystems; they are vital to their structure and functioning and are referred to as keystone species [1, 2]. For human populations worldwide, some species are also tightly woven into the fabric of society [3, 4]. These have been described as cultural keystone species, defined as “culturally salient species that shape in a major way the cultural identity of a people. Their importance is reflected in the fundamental roles these species play in diet, materials, medicine, and/or spiritual practices” [4]. This concept has since expanded to include cultural keystone place*s* [5, 6] and, more recently, spiritual keystone species [7]. Despite several other terms emerging in the scientific literature expressing similar and overlapping concepts (e.g., culturally important species [8], biocultural keystone species [9], ethnobiological keystone species [10]), the role of cultural keystone species in advancing conservation paradigms continues to be highlighted [11].

One important criterion for identifying cultural keystone species is the multiplicity of their uses. One can surmise that some species may be considered more culturally relevant if they are used for a wider range of purposes (e.g., dietary, technological, medicinal, spiritual, etc.). Within each of these domains, further subcategories of use may arise, enhancing the species’ cultural relevance. Building on these insights, it is timely to propose an extension of this framework to introduce another iteration of the concept: medicinal keystone species. Just as spiritual keystone species link cultural and spiritual practices to biodiversity [7], medicinal keystone species bridge cultural, ecological and health dimensions under a One Health framework, defined as an integrated approach that recognizes the interconnectedness and interdependence of human, animal, plant, and ecosystem health [12–14]. Medicinal keystone species may be defined as species that are disproportionately important to local medicinal systems, and whose decline, degradation or loss, including habitat, would represent a triple threat to human health and well-being, cultural identity, and biodiversity. In this context, they constitute a practical means of operationalizing One Health through the identification of salient species.

Although medicinal uses are considered within the broader scope of cultural keystone species, local medicinal systems exhibit structural and functional characteristics that warrant specific analytical attention. Cultural keystone species derive their importance from any of various uses (e.g., symbolic, dietary, material, spiritual). On the other hand, medicinal keystone species focus on therapeutic versatility, and explicitly reflect the logical foundations of a medicinal system. Rather than fragmenting existing analytical and conceptual frameworks, medicinal keystone species can be understood as a domain-specific adaptation that enhances the analytical resolution of cultural keystone species when the focus is on local health and medicinal systems.

Formalizing this concept underscores the need to recognize, study, and protect the species that are integral to both the social-ecological systems they inhabit and the medicinal systems they support. In a world increasingly threatened by biodiversity loss, such an approach not only enriches the understanding of the interdependence between human health and social-ecological systems, approaching the concept of reciprocity defined by Mattalia et al. [11], but also underlines the nexus between their social and ecological aspects and highlights the urgency of preserving natural resources that sustain global health.

## Description of concept

Among the six criteria describing a cultural keystone species [4], the first one specifies the multiplicity of uses, implying that medicinal keystone species fit as a subcategory alongside spiritual keystone species. In this case, a specific domain of use may acquire such structural and functional centrality that it becomes an organizing axis of the relationship between a human group and a given species in certain socioecological contexts. This is particularly evident in local medical systems, which are not simple aggregations of therapeutic practices but complex adaptive systems (see Albuquerque et al. [15]), suggesting that medicinal use often operates as an important component of broader socioecological dynamics. Under these circumstances, it becomes important to recognize medicinal keystone species as a subcategory of cultural keystone species due to their systemic relevance. As a sub-concept of cultural keystone species, the key criteria to define a medicinal keystone species, therefore, are the following:

**Table:** **Box 1**. Key criteria of medicinal keystone species

1.	Intensity, type, and diversity of medicinal use: captures the multiplicity, variability and extent of a species’ use.
2.	Naming and terminology in a language: records the diversity of terms used to name a species or associated with it, including seasonal, phenological, and/or biogeographical references.
3.	Role in narratives, ceremonies, and symbolism: reflects cultural and historical significance and embedded knowledge, and its contribution to group identity, particularly when the species serves as a symbolic organizing element of local healing logic.
4.	Persistence and memory of use in relationship to changes and threats: indicates continuity of use despite external and/or internal pressures on the local medicine system.
5.	Visibility and intelligibility of the foundations of a medicinal system: shows how a species’ use reflects the structural pillars and core principles of the local medicinal system.
6.	Opportunities for resource acquisition from beyond the local territory: considers the extent to which access, use, and mobility of a species increases capital (e.g., economic, social, cultural, natural, etc) through means like trade, commerce, and gifting.

Rather than regarding health, biodiversity and culture as separate domains, medicinal keystone species provide clear and distinct criteria through which their interdependence can be documented and assessed under a One Health perspective. Stating that an organism is a medicinal keystone species says more than merely articulating that an organism is an important medicinal resource. Medicinal keystone species are a well-defined category and an argued thesis based on the evaluation of specifically detailed criteria. It implies that a given species has been assessed rigorously against all criteria and that these are all met. As of yet, however, standardized methods or approaches for assessing medicinal keystone species (or cultural keystone species and places) remain underdeveloped, and a practical guide for operationalizing these criteria, including possible approaches to quantification, is essential for developing the concept. We therefore adopt a concept-building approach based on a preliminary qualitative and selected bibliographic assessment of (i) *kwachi* (*Quassia amara* L.; Simaroubaceae), a medicinal plant found in French Guiana (South America) and (ii) the beaver (*Castor canadensis* Kuhl, 1820; Castoridae), a medicinal animal present across North America. These examples are used to illustrate how these can be characterized as medicinal keystone species among French Guianese Creoles and Algonquian Peoples of Eastern Canada.

### Case 1: *Quassia amara*, a medicinal keystone plant

1. Despite its notable uses as a tonic, vermifuge, febrifuge and anti-malarial, *Q. amara* can be considered a culturally relevant panacea to several populations of French Guiana and afro-descendant populations throughout the Americas due to its multiple uses [16].
2. Its names in French-based Creoles (i.e. *quinine caraïbe, quina pays, quinquina de Cayenne* and *kwachi*), but also English-based Creoles (i.e. *kwasi*) not only serve as biogeographic indicators of the plant’s South American origin, but also of the role Afro-descendants played in the diffusion of its medicinal uses, i.e., one of the plant’s names bearing that of an enslaved African born in Guinea, Kwasi. This name also happens to have lent itself to the scientific genus (i.e., *Quassia* spp.) [16].
3. Biopiracy claims against the patenting of a molecule isolated from the leaves and bioactive against malaria and cancer were rooted in narratives linking this plant to cultural identity and the history of slavery [16]. Although the plant has naturalized itself in French Guiana, it occurs in former places of slavery (e.g., plantations and Maroon camps), where it continues to be harvested.
4. If such narratives help this plant survive the test of time, its relevance was highlighted during the COVID-19 pandemic; against the background of antivax sentiments, *Q. amara* became the treatment of choice [17, 18].
5. The plant’s bitter attributes are inextricably linked to the humoral system that marks a fundamental pillar of Creole medicine systems [17, 18].
6. *Q. amara* is sold on local markets, providing opportunities for the acquisition of financial resources, and circulates widely over the region, where it contributes to the cross-cultural hybridization of phytotherapies through the exchange of plants [19].

### Case 2: *Castor canadensis*, a medicinal keystone animal

1. The beaver (*C. canadensis*) is a prominent component of Algonquian food systems and, beyond its dietary role, is also valued medicinally - beaver castoreum is notably used to treat cuts and infections of all sizes and manners [20–23]. Due to the diverse medicinal properties ascribed to beavers and their multiple uses, this animal as a whole can also be considered a culturally relevant panacea to the numerous Algonquian First Nation groups
2. Names designating the beaver vary across different languages of the Algonquian language family reflecting dialectal differences [25, 26]. Nonetheless, the wide diversity of terms associated with this animal (roughly 130 terms in both the Eastern James Bay Cree Dictionary and Innu dictionary) bears witness to a comprehensive understanding of beaver biology and physiology, as well as its relationship with the environment, thus playing an important role in Algonquian ecological knowledge (e.g. *amiskusaakahiikan *meaning “a lake where there are beaver every year” and *uniipishtihchaau* meaning “a temporary summer beaver lodge”; Junker et al., 2018).
3. The beaver is prominently featured in Algonquian myths and appears in ceremonial and communal feasts [24].
4. Land-based healing programs and approaches to well-being that centre on traditional activities, including but not limited to hunting, traditional diets, and zootherapies, are increasingly being developed in Algonquian communities across Canada, e.g. [27–29]. These programs, such as the *Amisk* (Beaver) Harvesting Program in Subarctic Ontario [27], are grounded in discourses and actions regarding Indigenous rights to self-management, self-determination, and self-governance, which are rooted in the colonial history of North America.
5. The beaver’s wide-ranging medicinal uses are grounded in holistic healing concepts that consider and balance physical, spiritual, mental, and emotional health, core principles of several Algonquian medicine systems [24].
6. Beaver pelt has historically been a distinguishing feature of Canada’s fur trade [30]. Beaver trapping continues to be an important dietary subsistence activity, and various parts of the animal, such as the pelt, are still valorised for economic and artisanal purposes. Beaver meat continues to be served at community feasts and other publicized events, such as pow-wows, whereby non-Indigenous peoples are invited to learn about Algonquian culture and customs. Such events facilitate exchanges (e.g., cultural and material) in an environment that fosters cultural affirmation [24].

Altogether, these criteria highlight the level of unique position a given species has in its medicinal system. They reveal how it is difficult for them to be replaced with other species, whether they are autochthonous to these systems or not.

## Benefits of the concept

By specifically considering the foundational component of medicinal systems (i.e., criterion 5; **Box 1**), medicinal keystone species complement the broader concept of cultural keystone species by providing greater analytical resolution when focusing on health systems. Medicinal keystone species, when tied to the concept of cultural keystone places, may also play an important role in the conservation of social-ecological systems, especially if they are linked to the harvesting and acquisition of said species. Indeed, the cultural keystone places concept specifies 10 criteria, such as ecological diversity, including identified cultural keystone species [5], and by implicit extension, medicinal keystone species.

In fact, the concept was explicitly invoked in expert reports submitted to federal environmental review panels assessing the proposed New Prosperity Mine on the site of Teẑtan Biny (Fish Lake) in the Canadian province of British Columbia, which was characterized as a cultural keystone place containing several cultural important species of material, dietary, and medicinal importance to the Tŝilhqot’in First Nation, including beaver and several wild berries [5, 31, 32]. This evidence contributed to findings of significant adverse effects on Indigenous traditional land use and cultural practices [33], which were central to the Canadian government’s decision to reject the mining project [34]. This example highlights the heuristic, rather than strictly quantitative, role of cultural keystone places and the species associated with them in aiding conservation efforts, facilitating cross-cultural understanding in co-management, and helping to maintain a group’s cultural integrity and resilience [5, 35]. If medicinal keystone species inherently contribute to achieving these roles, they also contribute to increasing the recognition of local and traditional medicines in healthcare systems aiming to achieve universal health coverage.

Indeed, medicinal keystone species could inform public health policy by aligning with initiatives such as the WHO Global Centre for Traditional Medicine [36], which aims to support regulation and integrated health systems. In this way, they act as both ecological and health policy levers, providing a basis for conservation and healthcare decisions that strengthen universal health coverage while reinforcing the link between cultural and ecological systems. The concept may also provide a practical framework that contributes to operationalizing global diversity commitments, such as those made in the Kunming-Montreal Global Diversity Framework (KMGBF) with consideration of a One Health perspective [37]. In particular, Targets 5 and 9, which call for sustainable, safe and legal harvesting, use and trade of wild species while ensuring that their management delivers social, economic and environmental benefits, especially for biodiversity-dependent populations, prevents overexploitation, minimizes impacts on non-target species and ecosystems, and reduces the risk of pathogen spillover [37]. Based on its clearly defined criteria, medicinal keystone species translate One Health principles into practice by identifying salient species that may be addressed and prioritized in conservation planning and public health policies. Moreover, they may contribute to providing practical and operational means of addressing persistent gaps (see DeRoy et al. [38], Lukawiecki et al. [39], Oliva et al. [40], Sterling et al. [41]) between the formal recognition of IPLC in global diversity and health policy and the incorporation of their sociocultural conditions and characteristics into conventional monitoring and evaluation frameworks and platforms, such as KMGBF and the Intergovernmental Science-Policy Platform on Biodiversity and Ecosystem Services (IPBES).

The concept recognizes the medicinal knowledge systems of IPLC and emphasizes the key components of their *materia medica.* This is useful for the efficient implementation and access to local and traditional medicines in healthcare systems, particularly in areas with limited access to biomedical services that rely on local medicines as a first line of defence against disease, ailment or injury, as well as for maintaining overall good health and well-being. Due to their centrality in local medicinal systems, medicinal keystone species are particularly important to highlight for public health and to study from an ethnopharmacological point of view. If it is important to understand the mechanisms of action of a given species, it might also be evaluated for toxicity, as suggested recently [42]. Moreover, medicinal keystone species add value beyond existing frameworks by explicitly integrating structural, functional, cultural, and therapeutic dimensions of medicinal plant use within local medical systems, reflecting the socioecological nature of these systems. For example, the proposed concept is intricately tied to the notion of the structural core of medical systems, where there exists a set of resources (such as medicinal plants) that are disproportionately important and regulate the structure and functionality of local medical systems over time [43]. These ressources possess essential adaptive characteristics for medicinal use. By linking these two concepts, a medicinal keystone species can be identified as a species that, while part of the structural core, holds an even more prominent position. Although such species (i.e. plants and/or animals) within the structural core of medicinal systems may still undergo some degree of substitution over time, albeit at a slower pace than medicinal ressources from outside the core [43], the exclusion of a medicinal keystone species could threaten the integrity of the entire system. In addition to possessing the important characteristics that regulate the structure and functionality of the local medical system, they are also crucial resources for maintaining local cultural identity. To put it succinctly, medicinal keystone species are a restricted group of resources that fulfil structural and functional roles in both local medicinal systems and the cultural identity of peoples and society. The medicinal keystone species are identified through criteria that measure therapeutic versatility (criterion 1), cultural salience (criteria 3 and 4), structural position within the medicinal system (criterion 5), and low substitutability when considering all criteria together (**Box 1**). In this sense, medicinal keystone species contribute to the One Health perspective by bringing attention to the cultural and functional foundations of community health and well-being that depend on particular medicinal and natural resources. Given all the criteria considered in establishing the uniqueness of medicinal keystone species, they are most likely indicative of a form of pharmacological activity, regardless of whether or not we know how to evaluate it.

## Conclusion

If identified medicinal keystone species play a role in the conservation efforts of plant, fungal or animal species and their habitats, the concept may also promote sustainable harvesting practices informed by local and traditional ecological knowledge. Indeed, these species depend on the broader ecosystem that supports them. The preservation and conservation of their habitats and their sound management are crucial to ensuring the continued availability of local medicines. This highlights the interdependence of ecosystem health and human health and well-being. The “keystone species” concepts therefore capture the essence of the One Health concept, which emphasizes the links between plant, animal and human health [12–14], especially in its ability to cross disciplinary boundaries. Originating from the ecological keystone species concept, which is central to assessing ecosystems, medicinal keystone species come full circle to link the health of the environment and its species with human health and well-being. By providing grounded criteria for their identification, they offer a practical means for operationalizing One Health approaches in guiding as well as informing both conservation and public health research and policy.

## Data Availability

No datasets were generated or analysed during the current study.
